# Sympathy

**Published:** 1887-01-01

**Authors:** 


### Words of Consolation.

[Communications for this column should be addressed, "Chaplain," care of the Editor of The Hospital, who will gratefully welcome any suggestions or inquiries from matrons, nurses, and the staff generally.]

### XIV.—Sympathy.

There is another important consideration concerning Sympathy. We are members of one body, the Church of Christ. The better we are able to sympathise with one another, the more useful and really capable members do we become, however infirm we may be physically.

Here may be discerned another distinct purpose in Christian suffering, viz.: to teach us to sympathise. Suppose even your time in hospital is exhausted, and little or no progress made towards recovery. Suppose your physicians have prescribed for you, or operated in vain; and you have to go back home with the sad thought deeply graven that you may never be much better. Nevertheless, you have not entered the school of suffering for nought, if you have learnt some sympathy. You may go home with much tribulation in prospect, and yet with the cheerful assurance of becoming a more useful member of society than you ever were before—only in another way. Who are really the most valuable members of society? Those who have never known an ache or pain! Those who have always been so active, and hard-working, and energetic! They may seem so. We venture to think that Christians who have passed through tribulation, and have come forth capable of exercising their Priesthood in sympathy with their sorrowing or suffering brothers and sisters, are quite as necessary. "Nay, much more those members of the body which seem to be more feeble are necessary" (1 Cor. xii, 22). These are very scarce, and precious in homes where the physically or intellectually powerful are plentiful enough. Only see persons who have been blessed with health and strength for many a year laid up for the first time. What a difference it makes to them having some one to wait upon them who is no stranger to sickness! While the uninitiated are so careless about noises, or causing a great deal more worry than comfort with their "Never mind, you will soon be better," or "Keep up your spirits," the true sympathiser proves a friend indeed. Depend upon it, suffering is a great power for good if it does a little to deepen our feelings for our fellow-creatures as we see them in turn bound to submit to the great mystery of pain. How much we have to be thankful for if we find that a turn of sickness has softened us, and made us more considerate towards others when they are unwell. We can understand the murmurs we once thought so foolish, and make allowances for fancies and fidgets we once ridiculed. We become more like the Good Samaritan, because we can go near enough to see and grasp the fact that a fellow-creature is wounded: whereas before, we passed by on the other side, scarcely believing that he had much to complain of. How gladly would we avoid pain altogether if we could! Yet, if we would be disciples of Christ, we can desire no such thing at present. We may gratefully avail ourselves of any remedial agency God may place within our reach; and yet believe, or even desire, that He would have us suffer enough to prevent us ever thinking too lightly of what others have to bear. It is a very happy thing for us whenever we are touched with a feeling of their infirmities. There is no bond like Sympathy.

*(To be continued.)*

